# Editorial: postharvest ripening, senescence, and technology, Volume II

**DOI:** 10.3389/fgene.2026.1763492

**Published:** 2026-01-15

**Authors:** Angelos Deltsidis, Tie Liu, Karin Albornoz

**Affiliations:** 1 Department of Horticulture, University of Georgia, Tifton, GA, United States; 2 Horticultural Sciences Department, University of Florida, Gainesville, FL, United States; 3 Food, Nutrition, and Packaging Sciences Department, Coastal Research and Education Center, Clemson University, Charleston, SC, United States

**Keywords:** anthocyanins, fresh-cut, genomics, marker-assisted selection, quality, shelf-life, vase life

## Introduction

Fresh fruits, vegetables, and ornamentals are highly perishable. Once harvested, their quality and shelf-life rapidly decline, promoting consumer dissatisfaction, leading to food loss and waste, and jeopardizing the global goal of achieving food security. This Research Topic assessed how innovations in harvest management, postharvest technologies, and genomics-based breeding can improve quality traits. Shorter harvest intervals have been used to enhance blueberry postharvest quality (Godara et al.); pre- and postharvest approaches have been evaluated on their effectiveness in maintaining fresh-cut watermelon quality (Quandoh and Albornoz); multi-omics has elucidated volatile formation in pepper (Liu et al.); advanced genomics and genome editing have been applied to improve strawberry (Vondracek et al.); novel loci for canary yellow have been identified in watermelon (Park et al.), and genotype-environment-management interactions have been investigated to optimize lisianthus vase life and quality (Kim and Heo). Together, these studies illustrate how targeted pre- and postharvest strategies can optimize quality traits across diverse horticultural crops and contribute to food loss and waste reduction.

### Small fruit

Blueberry (*Vaccinium* spp.) fruit ripen asynchronously, which has direct effects on harvest timing and frequency. In a 2-year study, Godara et al. evaluated the effect of different picking intervals on quality attributes of rabbiteye blueberries (*Vaccinium virgatum*) “Brightwell”. Berries harvested every 7 days had higher fresh weight loss, berry damage, and softer texture than those from two- or 3-day intervals, indicating poorer storability. In contrast, longer, 7-day intervals produced berries with higher total soluble solids and anthocyanins, and lower titratable acidity levels, all of which suggest better suitability for processing, while the 3-day interval best balanced quality traits for the fresh market.

The allo-octoploid and highly heterozygous genome of strawberry (*Fragaria* × *ananassa*) makes it a challenging crop for conventional breeding. Vondracek et al. reviewed modern tools to study traits of interest at both agronomic/production and fruit quality levels. Marker-assisted selection (MAS) with techniques such as genomic selection, high-resolution melting (HRM) and simple sequence repeat analysis (SSR), together with DNA markers and rapid high-throughput DNA extraction methods, has supported breeding programs in developing improved cultivars. This was made possible by the wide range of genomic resources available for strawberry, along with the release of high-quality genome assemblies, which helped identify candidate genomic regions and genes associated with important traits. QTL mapping, genome-wide association studies (GWAS), and omics approaches (transcriptomics and metabolomics) have contributed to the innovations made in gene identification. Progress made in functional approaches, including overexpression, RNA interference, and CRISPR/Cas editing, is enabling the direct testing and manipulation of candidate genes, accelerating the release of new, improved cultivars.

### Watermelon

Watermelon (*Citrullus lanatus* L.) flesh color is determined by carotenoid composition and multiple interacting loci. Park et al. first described C_2_, a genetic determinant that influences yellow flesh color. Some canary-yellow lines carrying the classic dominant *C* allele at the lycopene β-cyclase locus still display an “incomplete” red–yellow phenotype, prompting further genetic analysis. Whole-genome resequencing and linkage mapping localized C_2_ to chromosome 2 near a pentatricopeptide repeat (PPR) gene, ClPPR, and marker analyses showed that full canary yellow color requires dominant alleles at both C and C_2_, while a recessive homozygous genotype produces red flesh irrespective of C_2_. The identified markers provide practical tools for MAS and underscore the epistatic control of carotenoid accumulation in cucurbits.

Fresh-cut processing favors convenience but also induces stress-related changes that reduce firmness, color, flavor, and antioxidant levels. Quandoh and Albornoz reviewed existing pre- and postharvest interventions aimed at extending fresh-cut watermelon shelf-life. Grafting can improve fruit firmness, while low-temperature storage is the key practice for quality retention. Processing conditions, chemical and natural antimicrobial compounds, and modified atmospheres have been the most studied, while novel treatments, such as edible coatings, have emerged in recent years. Overall, postharvest strategies were effective in maintaining certain quality traits but resulted in trade-offs in other aspects. The authors also highlighted the limited use of omics approaches focused on postharvest traits and the opportunity to develop cultivars specifically tailored to the fresh-cut market.

### Pepper

Volatile profiles influence consumer preferences in pepper (*Capsicum* spp.) quality; however, the underlying metabolic and genetic mechanisms are complex and mostly undiscovered. Liu et al. combined non-targeted metabolomics and transcriptome profiling during pepper fruit development to associate specific volatile compounds with changes that occur during lipid, amino acid, and terpenoid metabolism. Integrative network analysis highlighted candidate genes for fatty acid degradation, carotenoid cleavage, and terpene synthesis, as well as transcription factors potentially affecting flavor volatile accumulation. This work advances the understanding of the metabolic and transcriptional program interactions that create pepper aroma profiles and offers valuable molecular resources for strategies aimed at enhancing flavor quality in *Capsicum* species.

### Ornamentals

Lisianthus (*Eustoma grandiflorum*) vase life is influenced by genotype, growing conditions, and postharvest management. Kim and Heo evaluated four cultivars that were grown hydroponically or in soil, with or without salicylic acid (SA), applied at different growth stages and concentrations, to determine their effect on postharvest vase life. Hydroponic growing conditions with SA treatment during the reproductive stage resulted in vase life extension. Dry weight and the interaction between petal count and petal size were good predictors of vase life, with larger petals increasing longevity when petal count was low but reducing it when petal count was high. Overall, the study showed that lisianthus vase life extension requires genotype-specific growing conditions, carefully timed SA management, and a balanced floral morphology, highlighting the importance of integrated pre- and postharvest techniques for high-quality cut flowers.

## Conclusion

Across these investigations and reviews, precise harvest scheduling, appropriate postharvest technologies, and genomics-enabled breeding are effective approaches to enhance storability, sensory attributes, and overall market value of specialty crops. Challenges associated with climate change demand higher resource efficiency and underscore the need to explore innovative approaches to dissecting the basis of complex postharvest traits. Real-time monitoring of quality parameters through sensors, spectral imaging, and high-throughput technologies, combined with deep learning or machine learning tools, can expedite decision-making and facilitate the prediction of quality outcomes.

